# Improved genome assembly of *Candida auris* strain B8441 and annotation of B11205

**DOI:** 10.1128/mra.00512-24

**Published:** 2024-08-23

**Authors:** Nicholas C. Cauldron, Terrance Shea, Christina A. Cuomo

**Affiliations:** 1 Department of Molecular Microbiology & Immunology, Brown University, Providence, Rhode Island, USA; 2 Broad Institute of MIT and Harvard, Cambridge, Massachusetts, USA; University of California Riverside, Riverside, California, USA

**Keywords:** *Candida auris*, genomes, mycology

## Abstract

*Candida auris* is a fungal pathogen of significant worldwide concern, typically resistant to one or more antifungal drugs. We report a completed genome for clade Ia isolate B8441 and gene annotations of clade Ic isolate B11205. These resources will support public health investigations and population genomic studies of this pathogen.

## ANNOUNCEMENT

*Candida auris* is a fungal pathogen of worldwide concern, causing invasive disease in immunocompromised and critically ill patients (1). Unlike most fungi, most *C. auris* isolates are resistant to at least one class of antifungals, with common multidrug resistance (2). We completed the genome of *C. auris* clade Ia isolate B8441, a frequently selected reference in genomic studies (3–8). We transferred the gene annotations and added genes in filled-in gaps to provide an updated gene set. In addition, we transferred the gene set to the clade Ic isolate B11205, which may be a preferred reference as this isolate is more closely related to some outbreak clusters (3, 6).

We improved the previous B8441v2 genome from 15 scaffolds to seven chromosomes using alignment with the existing sequence to fill gaps (Table 1). First, scaffolds were aligned to each other using nucmer (--maxmatch and --nosimplify) from MUMmer v3.23 (9). We removed two small, redundant scaffolds (10.6 and 20.7 Kb) and placed another scaffold (11.8 Kb) within a mis-assembled region of a larger scaffold where its ends overlapped. To obtain a finished assembly of seven chromosomes, we assembled previously generated (10) long-read sequencing data (Oxford Nanopore: SRR14008616, Pacific Biosciences: SRR3883469) with Canu v1.6 and aligned resulting contigs to the B8441v2 assembly to bridge contigs and fill gaps; joins were then confirmed by aligning reads using minimap2 v2.15 (11, 12). To determine chromosome completeness, we searched for telomeric and centromeric sequences. Using nucmer (--maxmatch and --nosimplify), the fungal telomeric motif (TTAGG[GA]) was found on all sequence ends, except for the beginning of chromosome 3. Each scaffold of B8441v2 previously identified with a centromere (13) was syntenic with one chromosome of B8441v3.

**TABLE 1:** Assembly and annotation statistics for the improved assembly and annotations of *C. auris* isolates B8441 and B11205

	B8441v2	B8441v3	B11205
Scaffolds	15	7	7
Total length (Mb)	12.37	12.41	12.41
Longest scaffold (Mb)	3.20	4.18	4.16
N50 (Mb)	1.08	2.31	2.37
GC %	45.21	45.22	45.22
Genes^a^	5585	5594	5604
GenBank accession	GCA_002759435.2	GCA_002759435.3	GCA_016772135.1

^a^ Gene counts in B8441v3 and B11205 were expected to be highly similar since the primary annotation source was a liftover of genes from B8441v2.

To annotate genes in the B8441v3 and B11205 assemblies, we transferred existing annotations and supplemented genes identified *de novo*. All genes from B8441v2 were lifted over to B8441v3 using Liftoff v1.6.3 with default parameters (14). Genes that were partial in v2 because they were near contig ends or gaps were joined or filled in v3 when possible. *Ab initio* gene predictions were generated using GeneMark-ES (15). tRNAs and rRNAs were predicted using tRNAscan and RNAmmer, respectively (16, 17). Genes that overlapped incomplete lifted-over genes were used to fix the errant coordinates, and genes that did not overlap were added by using a Python script (complement_gff.py) (18). We then lifted the complete gene set of B8441v3 over to the B11205 genome and curated it as described previously. To identify genes *de novo* using BRAKER1 (3, 6), we supervised the training of GeneMark-ET and AUGUSTUS with RNA-seq data (PRJNA445471) (3, 19–22). We combined these results with tRNA and rRNA predictions to curate gene structures. We assigned new gene IDs to B8441v3 and B11205 using AGAT v1.3.0. We annotated known protein domains of all genes with InterproScan v5.66–98.0 (23, 24). To improve accessibility on the NCBI, we included special NCBI attributes in the gene feature annotations. We preserved equivalent identifiers from the B8441v2 annotations lifted over in both B8441v3 (old_locus_tag) and B11205 (note), facilitating use of previous literature referencing the v2 gene set. We used AGAT to add predicted functional information of protein domain matches from PFAM 36.0 and CDD 3.20 (db_xref) (25, 26).

**Fig 1:**
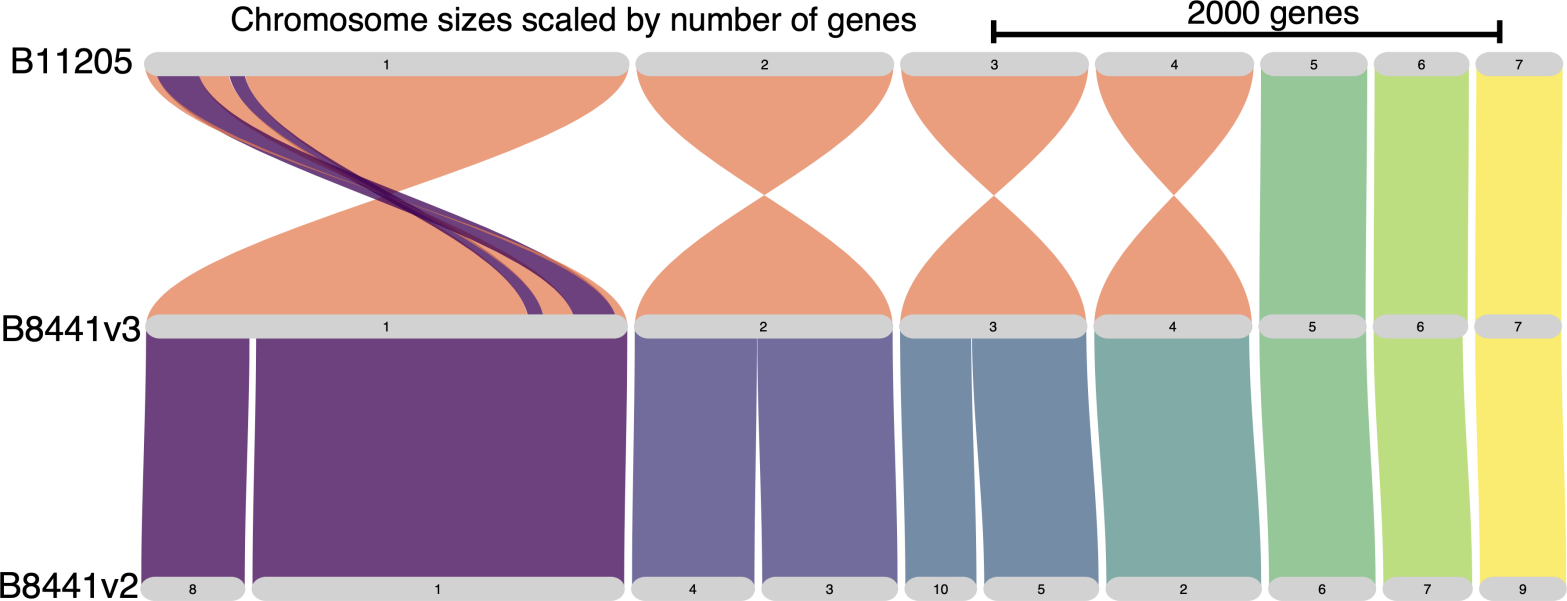
Homologs are highly collinear in all chromosomes of newly *C. auris* annotated genomes. The current reference genome (B8441v2) was finished into a near complete, chromosomal assembly (B8441v3). Scaffolds 10–15 of B8441v2 contained too few genes to visualize. Whole-chromosome inversions in B11205 are an artifact of assembly scaffold orientation (coral). Major differences between the two isolates are the two large inversions (>100 kbp) in chromosome 1 (purple).

We identified chromosomal rearrangements in B11205 using Minimap2 v2.26 (-f 0.0002) and GENESPACE v1.1.10 (27, 28). While all seven contigs were homologous between B8441 and B11205, two large regions are inverted in chromosome 1 (Fig. 1). One breakpoint of each inversion interrupted a protein-coding gene. The end breakpoint of the larger inversion interrupted CJI82_01928 (B8441v2: B9J08_001572), a putative transcription factor and homolog of *CTA3* in *C. albicans*. The starting breakpoint of the smaller inversion interrupted CJI82_01545 (B8441v2: not annotated), a putative methyltransferase and paralog of *TGS1* in *C. albicans*.

## Data Availability

The genome data reported here are available in GenBank under BioProject accession number PRJNA328792 and assembly accessions GCA_002759435.3 (B8441v3) and GCA_016772135.1 (B11205).

## References

[1] World Health Organization. 2022. WHO fungal priority pathogens list to guide research, development and public health action

[2] Chow NA, Gade L, Tsay SV, Forsberg K, Greenko JA, Southwick KL, Barrett PM, Kerins JL, Lockhart SR, Chiller TM, Litvintseva AP, US Candida auris Investigation Team. 2018. Multiple introductions and subsequent transmission of multidrug-resistant Candida auris in the USA: a molecular epidemiological survey. Lancet Infect Dis 18:1377–1384. doi:10.1016/S1473-3099(18)30597-8 30293877 PMC6556114

[3] Muñoz JF, Gade L, Chow NA, Loparev VN, Juieng P, Berkow EL, Farrer RA, Litvintseva AP, Cuomo CA. 2018. Genomic insights into multidrug-resistance, mating and virulence in Candida auris and related emerging species. Nat Commun 9:5346. doi:10.1038/s41467-018-07779-6 30559369 PMC6297351

[4] Chow NA, Muñoz JF, Gade L, Berkow EL, Li X, Welsh RM, Forsberg K, Lockhart SR, Adam R, Alanio A, Alastruey-Izquierdo A, Althawadi S, Araúz AB, Ben-Ami R, Bharat A, Calvo B, Desnos-Ollivier M, Escandón P, Gardam D, Gunturu R, Heath CH, Kurzai O, Martin R, Litvintseva AP, Cuomo CA. 2020. Tracing the evolutionary history and global expansion of Candida auris using population genomic analyses. MBio 11:e03364-19. doi:10.1128/mBio.03364-19 32345637 PMC7188998

[5] Salah H, Sundararaju S, Dalil L, Salameh S, Al-Wali W, Tang P, Ben Abid F, Tsui CKM. 2021. Genomic epidemiology of Candida auris in qatar reveals hospital transmission dynamics and a South Asian origin. J Fungi (Basel) 7:240. doi:10.3390/jof7030240 33807036 PMC8004815

[6] Muñoz JF, Welsh RM, Shea T, Batra D, Gade L, Howard D, Rowe LA, Meis JF, Litvintseva AP, Cuomo CA. 2021. Clade-specific chromosomal rearrangements and loss of subtelomeric adhesins in Candida auris. Genetics 218:iyab029. doi:10.1093/genetics/iyab029 33769478 PMC8128392

[7] Wang Y, Xu J. 2022. Population genomic analyses reveal evidence for limited recombination in the superbug Candida auris in nature. Comput Struct Biotechnol J 20:3030–3040. doi:10.1016/j.csbj.2022.06.030 35782746 PMC9218166

[8] Chowdhary A, Jain K, Chauhan N. 2023. Candida auris genetics and emergence. Annu Rev Microbiol 77:583–602. doi:10.1146/annurev-micro-032521-015858 37406342 PMC12962553

[9] Kurtz S, Phillippy A, Delcher AL, Smoot M, Shumway M, Antonescu C, Salzberg SL. 2004. Versatile and open software for comparing large genomes. Genome Biol 5:R12. doi:10.1186/gb-2004-5-2-r12 14759262 PMC395750

[10] Lockhart SR, Etienne KA, Vallabhaneni S, Farooqi J, Chowdhary A, Govender NP, Colombo AL, Calvo B, Cuomo CA, Desjardins CA, Berkow EL, Castanheira M, Magobo RE, Jabeen K, Asghar RJ, Meis JF, Jackson B, Chiller T, Litvintseva AP. 2017. Simultaneous emergence of multidrug-resistant Candida auris on 3 continents confirmed by whole-genome sequencing and epidemiological analyses. Clin Infect Dis 64:134–140. doi:10.1093/cid/ciw691 27988485 PMC5215215

[11] Koren S, Walenz BP, Berlin K, Miller JR, Bergman NH, Phillippy AM. 2017. Canu: scalable and accurate long-read assembly via adaptive k-mer weighting and repeat separation. Genome Res 27:722–736. doi:10.1101/gr.215087.116 28298431 PMC5411767

[12] Li H. 2018. Minimap2: pairwise alignment for nucleotide sequences. Bioinformatics 34:3094–3100. doi:10.1093/bioinformatics/bty191 29750242 PMC6137996

[13] Narayanan A, Vadnala RN, Ganguly P, Selvakumar P, Rudramurthy SM, Prasad R, Chakrabarti A, Siddharthan R, Sanyal K. 2021. Functional and comparative analysis of centromeres reveals clade-specific genome rearrangements in Candida auris and a chromosome number change in related species. MBio 12:e00905-21. doi:10.1128/mBio.00905-21 33975937 PMC8262905

[14] Shumate A, Salzberg SL. 2021. Liftoff: accurate mapping of gene annotations. Bioinformatics 37:1639–1643. doi:10.1093/bioinformatics/btaa1016 33320174 PMC8289374

[15] Lomsadze A, Ter-Hovhannisyan V, Chernoff YO, Borodovsky M. 2005. Gene identification in novel eukaryotic genomes by self-training algorithm. Nucleic Acids Res 33:6494–6506. doi:10.1093/nar/gki937 16314312 PMC1298918

[16] Lowe TM, Eddy SR. 1997. tRNAscan-SE: a program for improved detection of transfer RNA genes in genomic sequence. Nucleic Acids Res 25:955–964. doi:10.1093/nar/25.5.955 9023104 PMC146525

[17] Lagesen K, Hallin P, Rødland EA, Staerfeldt H-H, Rognes T, Ussery DW. 2007. RNAmmer: consistent and rapid annotation of ribosomal RNA genes. Nucleic Acids Res 35:3100–3108. doi:10.1093/nar/gkm160 17452365 PMC1888812

[18] Cox MP, Guo Y, Winter DJ, Sen D, Cauldron NC, Shiller J, Bradley EL, Ganley AR, Gerth ML, Lacey RF, McDougal RL, Panda P, Williams NM, Grunwald NJ, Mesarich CH, Bradshaw RE. 2022. Chromosome-level assembly of the Phytophthora agathidicida genome reveals adaptation in effector gene families. Front Microbiol 13:1038444. doi:10.3389/fmicb.2022.1038444 36406440 PMC9667082

[19] Hoff KJ, Lange S, Lomsadze A, Borodovsky M, Stanke M. 2016. BRAKER1: unsupervised RNA-seq-based genome annotation with GeneMark-ET and AUGUSTUS. Bioinformatics 32:767–769. doi:10.1093/bioinformatics/btv661 26559507 PMC6078167

[20] Lomsadze A, Burns PD, Borodovsky M. 2014. Integration of mapped RNA-Seq reads into automatic training of eukaryotic gene finding algorithm. Nucleic Acids Res 42:e119. doi:10.1093/nar/gku557 24990371 PMC4150757

[21] Stanke M, Diekhans M, Baertsch R, Haussler D. 2008. Using native and syntenically mapped cDNA alignments to improve de novo gene finding. Bioinformatics 24:637–644. doi:10.1093/bioinformatics/btn013 18218656

[22] Kim D, Pertea G, Trapnell C, Pimentel H, Kelley R, Salzberg SL. 2013. TopHat2: accurate alignment of transcriptomes in the presence of insertions, deletions and gene fusions. Genome Biol 14:R36. doi:10.1186/gb-2013-14-4-r36 23618408 PMC4053844

[23] Jones P, Binns D, Chang H-Y, Fraser M, Li W, McAnulla C, McWilliam H, Maslen J, Mitchell A, Nuka G, Pesseat S, Quinn AF, Sangrador-Vegas A, Scheremetjew M, Yong S-Y, Lopez R, Hunter S. 2014. InterProScan 5: genome-scale protein function classification. Bioinformatics 30:1236–1240. doi:10.1093/bioinformatics/btu031 24451626 PMC3998142

[24] Blum M, Chang H-Y, Chuguransky S, Grego T, Kandasaamy S, Mitchell A, Nuka G, Paysan-Lafosse T, Qureshi M, Raj S, et al.. 2021. The InterPro protein families and domains database: 20 years on. Nucleic Acids Res 49:D344–D354. doi:10.1093/nar/gkaa977 33156333 PMC7778928

[25] Marchler-Bauer A, Anderson JB, Cherukuri PF, DeWeese-Scott C, Geer LY, Gwadz M, He S, Hurwitz DI, Jackson JD, Ke Z, Lanczycki CJ, Liebert CA, Liu C, Lu F, Marchler GH, Mullokandov M, Shoemaker BA, Simonyan V, Song JS, Thiessen PA, Yamashita RA, Yin JJ, Zhang D, Bryant SH. 2005. CDD: a conserved domain database for protein classification. Nucleic Acids Res 33:D192–6. doi:10.1093/nar/gki069 15608175 PMC540023

[26] Mistry J, Chuguransky S, Williams L, Qureshi M, Salazar GA, Sonnhammer ELL, Tosatto SCE, Paladin L, Raj S, Richardson LJ, Finn RD, Bateman A. 2021. Pfam: the protein families database in 2021. Nucleic Acids Res 49:D412–D419. doi:10.1093/nar/gkaa913 33125078 PMC7779014

[27] Lovell JT, Sreedasyam A, Schranz ME, Wilson M, Carlson JW, Harkess A, Emms D, Goodstein DM, Schmutz J. 2022. GENESPACE tracks regions of interest and gene copy number variation across multiple genomes. Elife 11:e78526. doi:10.7554/eLife.78526 36083267 PMC9462846

[28] R Core Team. 2023. R: a language and environment for statistical computing. Vienna, Austria R Foundation for Statistical Computing

